# State-dependent dual-site prefrontal TMS bidirectionally modulates working-memory accuracy

**DOI:** 10.3389/fnhum.2026.1836043

**Published:** 2026-06-12

**Authors:** Jinwen Wei, Sida Chen, Werner Sommer, Changsong Zhou

**Affiliations:** 1 Department of Physics, Hong Kong Baptist University, Kowloon Tong, Hong Kong SAR, China; 2 Centre for Nonlinear Studies and Beijing-Hong Kong-Singapore Joint Centre for Nonlinear and Complex Systems (Hong Kong), Hong Kong Baptist University, Kowloon Tong, Hong Kong SAR, China; 3 Institute of Computational and Theoretical Studies, Hong Kong Baptist University, Kowloon Tong, Hong Kong SAR, China; 4 Institut für Psychologie, Humboldt-Universität zu Berlin, Berlin, Germany; 5 Faculty of Education, National University of Malaysia, Kuala Lumpur, Malaysia; 6 Life Science Imaging Centre, Hong Kong Baptist University, Kowloon Tong, Hong Kong SAR, China

**Keywords:** Bayesian analysis, dorsolateral prefrontal cortex, dual-site stimulation, medial prefrontal cortex, state-dependent effects, theta-burst stimulation, transcranial magnetic stimulation, working memory

## Abstract

Working memory (WM) is a core cognitive function that is both mechanistically tractable and clinically relevant, making it a prime target for noninvasive neuromodulation. However, transcranial magnetic stimulation (TMS) studies targeting the dorsolateral prefrontal cortex (DLPFC) have yielded variable WM outcomes, possibly because single-site protocols fail to engage the distributed network dynamics that support WM and because effects depend on momentary brain states. Here, we tested whether dual-site sequential TMS, excitatory intermittent theta-burst stimulation (iTBS) over left DLPFC combined with inhibitory continuous theta-burst stimulation (cTBS) over medial prefrontal cortex (mPFC), produces state-dependent modulation of WM performance compared with single-site DLPFC stimulation alone. Thirty-seven healthy adults completed a within-subjects protocol with intermixed 0-back and 2-back WM conditions. Self-reported fatigue before each task run served as a coarse indicator of the participant’s cognitive state. Bayesian linear mixed models revealed a clear state-dependent pattern: in the high-load 2-back condition, dual-site stimulation improved accuracy when participants reported low fatigue but impaired accuracy when they reported high fatigue. No reliable effects were found for the 0-back condition or for reaction time. These findings demonstrate that dual-site prefrontal TMS can bidirectionally modulate WM accuracy relative to single-site stimulation depending on the participant’s cognitive state, highlighting the importance of state-aware and network-sensitive stimulation approaches for cognitive enhancement.

## 1 Introduction

Working memory (WM), the capacity to temporarily maintain and manipulate information over brief intervals, is fundamental to goal-directed behaviors including reasoning, language comprehension, and decision-making (Baddeley, 2003; Chai et al., 2018). WM capacity is limited, varies across individuals, and is disrupted in numerous mental and neurological disorders such as schizophrenia, attention-deficit/hyperactivity disorder, and age-related cognitive decline (Barch and Ceaser, 2012). Because of its centrality to both normal cognition and clinical pathology, WM has become a primary target for cognitive enhancement interventions.

Transcranial magnetic stimulation (TMS) has emerged as a leading tool for probing the causal neural mechanisms of WM and for exploring the potential to enhance WM performance (Rossi et al., 2021). Among TMS protocols, theta-burst stimulation (TBS) has gained particular attention due to its ability to induce long-lasting aftereffects on cortical excitability with short stimulation durations. Intermittent TBS (iTBS) is generally excitatory, increasing cortical excitability, whereas continuous TBS (cTBS) is inhibitory (Huang et al., 2005). These properties make TBS protocols especially suitable for experiments requiring efficient modulation of multiple brain regions within a single session.

Most WM–TMS studies have focused on the dorsolateral prefrontal cortex (DLPFC), a key node in the frontoparietal executive-control network consistently activated during WM tasks (Wager and Smith, 2003; Curtis and D'Esposito, 2003). While excitatory TMS over DLPFC can modulate WM performance, effects are often modest and inconsistent (Asgarinejad et al., 2024; Xu et al., 2024), possibly reflecting differences in stimulation parameters, neuroanatomical variability, and the failure of single-site protocols to capture the distributed network dynamics underlying WM.

WM does not depend on the DLPFC in isolation but relies on the coordinated interplay between multiple large-scale brain networks (Liang et al., 2015). More broadly, dynamic interactions among distributed brain regions, including interhemispheric communication, have been shown to shape cognitive and psychiatric outcomes across development (Wu et al., 2025) and to support compensatory reorganization following brain injury (Fan et al., 2026), underscoring the general principle that cognitive functions emerge from network-level coordination rather than isolated regional activity. Effective WM performance is associated with activation of task-positive regions and concurrent deactivation of the default-mode network (DMN), particularly the medial prefrontal cortex (mPFC), a major DMN hub (Anticevic et al., 2012). Greater task-related suppression of mPFC activity has been shown to correlate with better WM performance, and reduced mPFC suppression is associated with diminished anticorrelation between mPFC and DLPFC (Whitfield-Gabrieli et al., 2009). These observations suggest that the balance between DLPFC activation and mPFC suppression is a critical determinant of WM efficiency. Given this network perspective, stimulating the DLPFC alone may be insufficient to produce robust WM enhancement. Multi-site stimulation approaches have been proposed to address this limitation by perturbing coordinated activity among interconnected regions (Wei et al., 2026). A dual-site approach combining excitatory stimulation of the DLPFC with inhibitory stimulation of the mPFC could reinforce the natural task-positive/DMN antagonism that supports effective WM performance.

The dual-site targeting is further supported by the emerging concept of network “sloppiness,” which posits that not all dimensions of neural network variation contribute equally to cognitive performance (Chen et al., 2025). In sloppy systems, performance depends disproportionately on variations along a small number of “stiff” dimensions—mechanistically influential axes of network activity—while being relatively insensitive to variations along “sloppy” dimensions. Applied to WM, this framework suggests that the DLPFC and mPFC occupy dissociable yet interacting positions along the stiff dimensions most relevant to WM control, and that perturbation along these stiff dimensions should have the greatest impact on behavior.

However, the effects of brain stimulation are unlikely to be uniform across all conditions. A growing body of evidence indicates that TMS outcomes are strongly influenced by the momentary state of the brain at the time of stimulation (Silvanto et al., 2008; Bradley et al., 2022). The same stimulation protocol can enhance, impair, or have no effect on performance depending on neural activity levels, arousal, and network configuration. This state dependency reflects the nonlinear dynamics of neural populations, in which the response to external perturbation depends on the baseline operating point of the system. Recent work demonstrates that large-scale cortical dynamics fluctuate along low-dimensional arousal-related manifolds that shape cognitive readiness and susceptibility to perturbation (Raut et al., 2025), potentially altering the functional connectivity between DLPFC and mPFC and thereby modulating the effectiveness of dual-site TMS.

Self-reported fatigue provides a behaviorally accessible window into these state fluctuations. Subjective fatigue ratings have been shown to covary with objective markers of reduced arousal, including decreased pupil diameter and diminished stimulus-evoked pupil responses during working memory performance (Hopstaken et al., 2015). At the neural level, subjective mental fatigue is associated with increased default-mode network activity during task engagement and concurrent hypoactivity of task-related regions (Gergelyfi et al., 2021), and with weakened anticorrelations between DMN nodes and task-positive cortex (Gui et al., 2015). These dynamics are directly relevant to WM, as the magnitude of mPFC–DLPFC anticorrelation has been shown to predict WM capacity in healthy adults (Keller et al., 2015). When fatigue is high, mPFC activity tends to persist rather than suppress during task engagement, and the anticorrelation between DLPFC and mPFC becomes less stable, precisely the network configuration that dual-site TMS targeting these two regions is designed to modulate. A low-fatigue state, by contrast, is associated with robust DLPFC engagement and effective mPFC suppression, creating conditions that may be receptive to further reinforcement through excitatory DLPFC and inhibitory mPFC stimulation. Moreover, fatigue is among the most commonly experienced subjective states during repetitive cognitive testing (Ackerman and Kanfer, 2009), making it both ecologically representative and practically accessible as a state indicator in within-session designs. Fatigue thus serves not merely as a nuisance variable but as a theoretically motivated proxy for the DLPFC–mPFC balance that determines how dual-site perturbation will interact with ongoing brain dynamics.

Together, these lines of evidence lead to a specific hypothesis: excitatory stimulation of the DLPFC coupled with inhibitory stimulation of the mPFC should produce stronger and more state-sensitive WM modulation than stimulating the DLPFC alone, with the direction of modulation depending on the participant’s cognitive state. We tested this hypothesis in a within-subjects experiment using sequential theta-burst stimulation and a well-validated WM task, with self-reported fatigue serving as a proxy for the participant’s momentary cognitive state.

## 2 Materials and methods

### 2.1 Participants

Forty healthy young adults were recruited from the local university community. All participants were right-handed, had normal or corrected-to-normal vision, had no history of neurological or psychiatric illness, no contraindications to TMS, and no current use of psychoactive medications. Written informed consent was obtained from all participants. The study was approved by the Research Ethics Committee of Hong Kong Baptist University in accordance with the Declaration of Helsinki. Three participants were excluded due to incomplete data, yielding a final sample of thirty-seven participants (eight males, mean age = 24.1 ± 3.7 years). The sample size was informed by comparable within-subjects TBS studies targeting the DLPFC during working memory tasks, which have typically employed 19 to 51 participants (Hoy et al., 2016; Li et al., 2025; Schicktanz et al., 2015). A formal frequentist power analysis was not conducted *a priori*, as inference was planned within a Bayesian framework that provides direct posterior probability statements about effect sizes without relying on pre-specified Type I error rates (Kruschke and Liddell, 2018).

### 2.2 Experimental design and procedure

Each participant attended two sessions separated by 7 days. The two sessions corresponded to: (i) a dual-site session with excitatory iTBS over left DLPFC followed by inhibitory cTBS over mPFC, and (ii) a single-site session with excitatory iTBS over left DLPFC followed by sham cTBS over mPFC (coil rotated 90°). Session order (dual-site first vs. single-site first) was fully counterbalanced across participants. The within-session stimulation order (iTBS-first vs. cTBS-first) was counterbalanced between subjects but held constant across the two sessions within each subject, so that any order-related effects would be consistent across stimulation conditions. Participants reported their sleep duration on the night before each session. Across all 74 session visits, the mean self-reported sleep duration was 6.9 ± 0.9 h (range: 5.0–8.0 h; median: 7.0 h), consistent with typical sleep patterns in young adult university populations.

**Figure 1:**
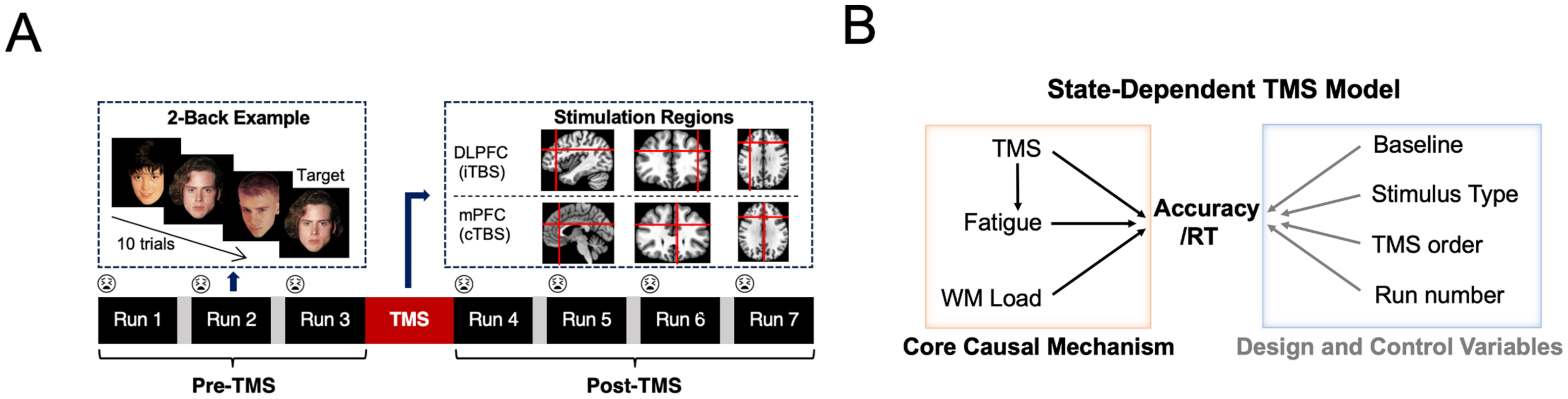
Experimental design and state-dependent TMS effect model. **(A)** Study timeline and working-memory task structure. Participants completed three pre-TMS runs and four post-TMS runs of an HCP-derived WM task containing intermixed 0-back and 2-back WM blocks with naturalistic stimuli (StimType: faces, places, tools, and body parts).The face stimulus images shown in the 2-back example in panel A were obtained from the HCP Young Adult 3T task-fMRI Working Memory stimulus/E-Prime materials, available in the public domain through ConnectomeDB (https://www.humanconnectome.org/hcp-protocols-ya-task-fmri). Fatigue state (Low/High) was reported before each run (face icon). Stimulation occurred between Runs 3 and 4. Each participant completed two sessions. In the single-site session, participants received active iTBS over left DLPFC paired with sham cTBS over mPFC (coil rotated 90°). In the dual-site session, they received active iTBS over DLPFC (MNI: −40, 34, 29) and active cTBS over mPFC (MNI: −4, 30, 30). Both iTBS and cTBS followed standard protocols (600 pulses, iTBS: 190 s, cTBS: 40 s), with stimulation delivered sequentially and an inter-protocol interval of approximately 30 s. All targets were individualized using each participant’s T1-weighted MRI. The session order was fully counterbalanced across participants, and the within-session stimulation order (iTBS-first vs. cTBS-first) was counterbalanced between subjects but fixed within each subject across sessions. **(B)** State-dependent TMS model. The core causal mechanism assumes that TMS influences WM performance directly and that the magnitude and direction of this effect vary with the participant’s momentary fatigue state and WM load. Additional design variables (baseline performance, stimulus type, run number, and stimulation order) were included as covariates.

Within each session, participants completed three pre-TMS and four post-TMS runs of the WM task (Figure 1A), with stimulation administered between Runs 3 and 4. Immediately before each run, participants reported their current fatigue level as “Low” or “High” via a single button press. Following this response, a brief instruction screen indicated the upcoming condition (0-back or 2-back), and the first trial requiring a response appeared approximately 2 s after the fatigue rating, minimizing the interval between state assessment and task onset. These ratings served as a coarse indicator of the participant’s cognitive state, reflecting fluctuations in arousal and attentional readiness that shape network dynamics and susceptibility to neuromodulation (Bradley et al., 2022; Wei et al., 2024).

### 2.3 Working memory task

WM was assessed using a task adapted from the Human Connectome Project (HCP) paradigm (Barch et al., 2013). The task comprised intermixed blocks of a low-load (0-back) and high-load (2-back) condition using naturalistic visual stimuli from four categories: faces, places, tools, and body parts (Figure 1A). In the 0-back condition, participants responded whenever the current stimulus matched a target shown at the beginning of the block. In the 2-back condition, participants indicated whether the current stimulus matched the one presented two trials earlier, requiring continuous WM updating. Each run contained equal numbers of 0-back and 2-back blocks with randomized order. Behavioral outcomes were accuracy and median reaction time (RT).

### 2.4 TMS protocol

TMS was delivered using a MagPro X100 system (MagVenture, Farum, Denmark) equipped with a figure-of-eight coil (CB-60). Prior to the experimental sessions, each participant’s resting motor threshold (RMT) was determined using standard electromyographic procedures by recording motor evoked potentials from the right first dorsal interosseous muscle. The mean RMT across participants was 43.6 ± 9.8% of maximum stimulator output (MSO; range: 22–60% MSO). Stimulation intensity for both iTBS and cTBS was set at 80% of each participant’s RMT, yielding a mean stimulation intensity of 34.9 ± 7.8% MSO (range: 18–48% MSO). The same intensity was used across both sessions within each participant, based on the RMT obtained in the first session. This intensity falls within the range commonly used in TBS research and is considered safe and tolerable for healthy adults (Rossi et al., 2021).

Both iTBS and cTBS followed standard published protocols (Huang et al., 2005). iTBS consisted of bursts of three pulses at 50 Hz repeated at 5 Hz, delivered in 2-s trains with 8-s inter-train intervals, for a total of 600 pulses over approximately 190 s. cTBS consisted of a continuous train of three-pulse bursts at 50 Hz repeated at 5 Hz for a total of 600 pulses delivered over 40 s. In the dual-site session, active iTBS was delivered over the left DLPFC, targeting MNI coordinates (−40, 34, 29) derived from a lesion-mapping study identifying the dorsolateral prefrontal region most consistently associated with working memory performance (Barbey et al., 2013). Active cTBS was delivered over the mPFC, targeting MNI coordinates (−4, 30, 30), adapted from a dorsomedial prefrontal stimulation target (0, 30, 30) used in a prior theta-burst stimulation study in depression (Struckmann et al., 2022); a 4 mm leftward shift was applied to provide clearance for attaching the optical tracker to the neuronavigation system without compromising stimulation of the mPFC target. In the single-site session, active iTBS was delivered over the same left DLPFC target, and sham cTBS was delivered over the mPFC with the coil rotated 90°. The coil was held tangentially to the scalp at each target site, with the handle oriented posteriorly for the DLPFC target. The two stimulation protocols were delivered sequentially with an inter-protocol interval of typically around 30 s, facilitated by real-time neuronavigation. All stimulation targets were individualized for each participant using neuronavigation guided by individual T1-weighted structural MRI scans to ensure anatomical precision.

Structural MRI scans were acquired on a 3 T Siemens MAGNETOM scanner (Siemens Healthineers, Erlangen, Germany) using a T1-weighted MPRAGE sequence with the following parameters: TR = 2,300 ms, TE = 2.28 ms, TI = 900 ms, flip angle = 8°, voxel size = 1.0 × 1.0 × 1.0 mm^3^, FOV = 256 × 256 mm^2^, matrix = 256 × 256, 192 sagittal slices, GRAPPA acceleration factor = 2, bandwidth = 200 Hz/pixel, acquisition time = 5 min 30 s.

### 2.5 Statistical analysis

To estimate the state-dependent causal effects of stimulation on WM performance, we fit Bayesian linear mixed models separately for accuracy and median RT. The models included the following specification:


$$\begin{array}{l} \text{Accuracy}\;(\text{or}\;\mathrm{RT})\sim \mathrm{TMS}\times \mathrm{WM}\;\text{Load}\times \text{Fatigue}+\text{Baseline}\\ +\mathrm{Run}+\text{StimType}+\mathrm{TMS}\;\text{Order}+(\begin{array}{l} 1+\mathrm{TMS}\times \mathrm{WM}\;\text{Load}\\ \times \text{Fatigue}\;|\;\text{Subject} \end{array}). \end{array}$$

The three-way interaction TMS × WM Load × Fatigue represents the core mechanism of interest (Figure 1B), capturing how dual-site versus single-site stimulation effects on WM vary by cognitive load and fatigue state. All other predictors were precision-improving covariates: Baseline controlled for pre-stimulation performance, Run for practice or fatigue trends, StimType for stimulus category differences, and TMS Order for the counterbalanced within-session stimulation order (iTBS-first vs. cTBS-first). Random slopes for the three-way interaction accommodated individual differences in sensitivity to stimulation and fatigue.

To examine whether the within-session stimulation order (iTBS-first vs. cTBS-first) influenced the state-dependent effects, we fitted a supplementary Bayesian linear mixed model on the 2-back accuracy data only, replacing WM Load with TMS Order in the three-way interaction:


$$\begin{array}{l} \text{Accuracy}\sim \mathrm{TMS}\times \mathrm{TMS}\;\text{Order}\times \text{Fatigue}+\text{Baseline}\\ +\mathrm{Run}+\text{StimType}+(\begin{array}{l} 1+\mathrm{TMS}\times \mathrm{TMS}\;\text{Order}\\ \times \text{Fatigue}\;|\;\text{Subject} \end{array}). \end{array}$$

This model directly tests whether the state-dependent dual-versus-single contrast differed depending on whether excitatory DLPFC stimulation (iTBS) or inhibitory mPFC stimulation (cTBS) was delivered first within each session. Posterior summaries were computed as in the primary analysis.

Bayesian estimation used weakly informative priors to regularize parameter estimates while allowing the data to drive inference (Franke and Roettger, 2019). Effects were considered robust when the 95% highest posterior density (HPD) interval excluded zero and at least 97.5% of posterior probability (*Pr*) supported the effect direction. This Bayesian approach provides natural probabilistic interpretation of estimates and uncertainty, accommodates hierarchical data structure, and avoids difficulties with *p*-values and multiple comparison corrections (Kruschke and Liddell, 2018).

## 3 Results

**Figure 2:**
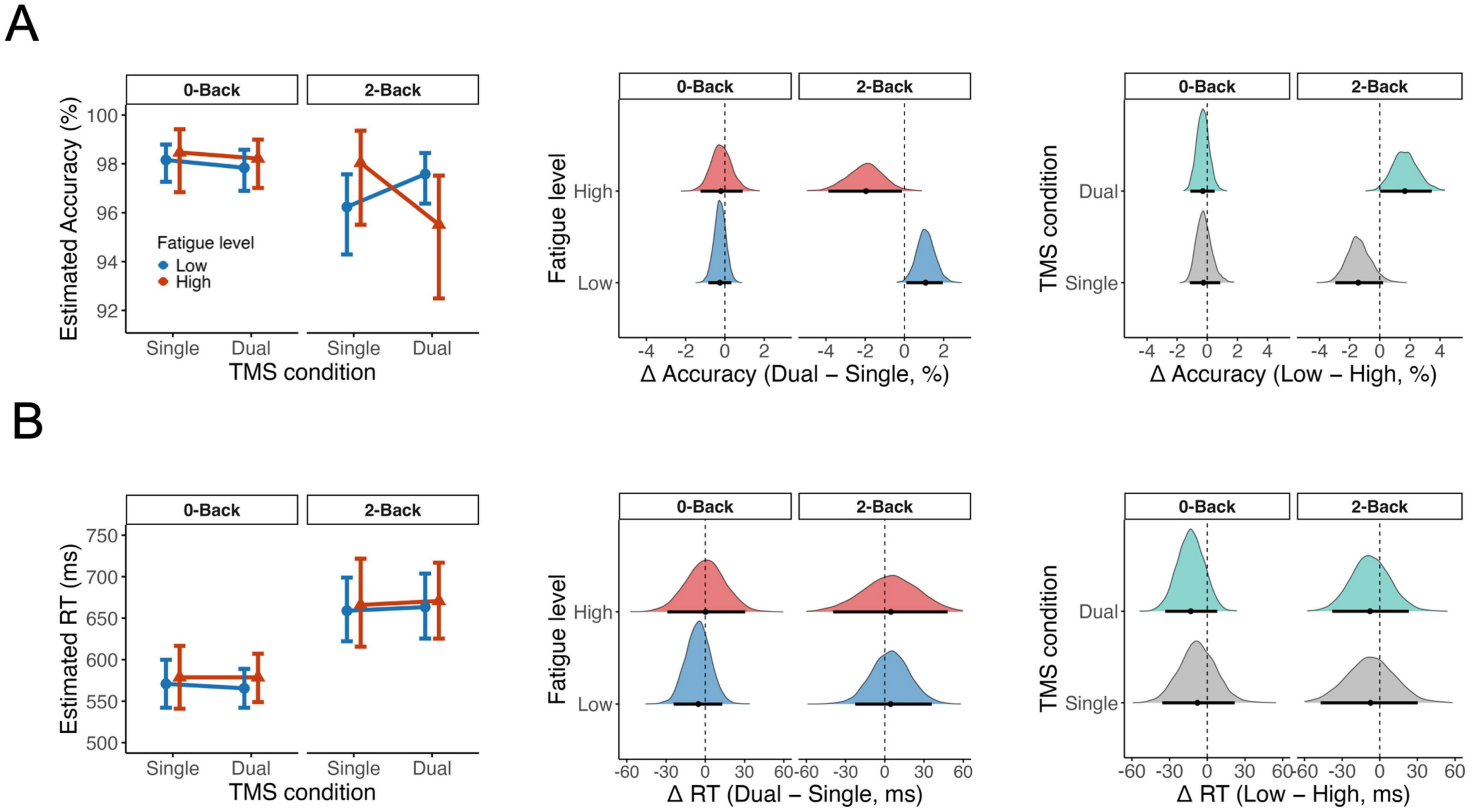
Bayesian behavioral results. **(A)** Posterior accuracy estimates. Left: Bayesian model estimates for single- and dual-site stimulation across fatigue states. Middle: Posterior distributions of *Δ*Accuracy (Dual − Single). Right: Posterior distributions of ΔAccuracy (Low − High). A robust fatigue-related difference was observed selectively in the 2-back dual-site condition (median ΔAccuracy for Low − High = 1.66, 95% HPD [0.02, 3.45%], *Pr*(Δ > 0) = 0.98). **(B)** Posterior reaction-time (RT) estimates, showing no robust modulation across all comparisons.

A clear state-dependent pattern emerged from the Bayesian analysis (Figure 2). The critical three-way interaction between stimulation condition, WM load, and fatigue state was supported by the posterior distributions, revealing that the effect of dual-site versus single-site TMS on accuracy differed depending on both the cognitive load and the participant’s self-reported fatigue level.

In the high-load 2-back condition, dual-site stimulation significantly improved accuracy relative to single-site stimulation during runs in which participants reported low fatigue. The posterior median of the accuracy difference (Dual − Single) was 1.08%, with the 95% HPD interval ranging from 0.10 to 1.95%, and 99% of the posterior probability mass supporting a positive effect (*Pr*(*Δ* > 0) = 0.99). In contrast, when participants reported high fatigue, dual-site stimulation significantly impaired 2-back accuracy compared with single-site stimulation. The posterior median of ΔAccuracy was −1.96%, with the 95% HPD interval spanning from −3.87 to −0.13%, and 98% of the posterior probability supporting a negative effect (*Pr*(Δ < 0) = 0.98). These opposing effects produced a robust fatigue-related difference selectively in the 2-back dual-site condition: the posterior median of the accuracy difference between Low and High fatigue states (Low − High) was 1.66%, with the 95% HPD interval ranging from 0.02 to 3.45% and *Pr*(Δ > 0) = 0.98. For all other conditions, including the 2-back single-site condition and both fatigue levels of the 0-back condition, the corresponding HPD intervals for the Low − High fatigue difference included zero, indicating no reliable fatigue-dependent modulation.

In the low-load 0-back condition, no reliable differences between dual-site and single-site stimulation were observed regardless of fatigue state. The posterior medians of ΔAccuracy were close to zero, and the 95% HPD intervals were wide and spanned zero for both low-fatigue and high-fatigue runs, suggesting that dual-site stimulation did not meaningfully alter performance when WM demands were minimal.

Analysis of reaction times revealed no reliable effects of stimulation condition, WM load, or fatigue state. Across all comparisons, the posterior distributions of *Δ*RT (Dual − Single) were centered near zero with broad HPD intervals that included zero (Figure 2B). Similarly, the fatigue-related RT differences (Low − High) within each stimulation condition showed no robust modulation. These results indicate that the dual-site stimulation protocol did not affect general response speed but selectively influenced the accuracy of WM representations under high cognitive load.

A supplementary analysis examined whether the within-session stimulation order (iTBS-first vs. cTBS-first) modulated the state-dependent effects observed in the 2-back condition. A Bayesian linear mixed model was fitted on the 2-back accuracy data, replacing WM Load with TMS Order in the three-way interaction (see Section 2.5). The bidirectional pattern was qualitatively consistent across both stimulation orders: in both the iTBS-first and cTBS-first groups, dual-site stimulation showed a numerical accuracy advantage under low fatigue (iTBS-first: median ΔAccuracy (Dual − Single) = 0.84%, *Pr*(Δ > 0) = 0.95; cTBS-first: median ΔAccuracy = 1.24%, *Pr*(Δ > 0) = 0.98) and a numerical disadvantage under high fatigue (iTBS-first: median ΔAccuracy = −1.43%, *Pr*(Δ < 0) = 0.88; cTBS-first: median ΔAccuracy = −2.18%, *Pr*(Δ < 0) = 0.97). The direction of the state-dependent modulation was identical in both groups, indicating that the bidirectional effect was not driven by a particular stimulation order.

## 4 Discussion

The present study demonstrates that dual-site prefrontal TMS targeting both the DLPFC and mPFC does not exert uniform effects on WM performance but instead interacts with the participant’s momentary cognitive state to produce bidirectional modulation of WM accuracy. Specifically, when participants were in a low-fatigue state, combining excitatory DLPFC stimulation with inhibitory mPFC stimulation improved 2-back accuracy beyond the level achieved by DLPFC stimulation alone. Conversely, when participants reported high fatigue, the same dual-site protocol impaired 2-back accuracy. These effects were specific to the high-load WM condition and did not extend to response speed, suggesting that dual-site TMS selectively modulated the quality of WM representations rather than general processing efficiency.

Although the present study did not include concurrent neuroimaging, the opposing effects observed under low versus high fatigue are consistent with a mechanistic account grounded in the dynamic interplay between task-positive prefrontal systems and default-mode processes anchored in the mPFC (Wager and Smith, 2003; Anticevic et al., 2012). Under conditions of low fatigue or high alertness, the frontoparietal control network and the DMN are likely operating in their characteristic anticorrelated configuration, with DLPFC activity elevated and mPFC activity suppressed during task engagement. In this well-configured state, excitatory iTBS over DLPFC and inhibitory cTBS over mPFC may reinforce the existing task-positive configuration, effectively amplifying the natural network dynamics that support WM and pushing performance slightly higher. This interpretation aligns with the state-dependency principle in brain stimulation, which holds that TMS effects are most beneficial when they augment the neural activity pattern already engaged by the task (Silvanto et al., 2008).

Under high fatigue, however, the control state of the brain is likely less stable and less optimally configured for task performance. Fatigue is associated with reduced sustained attention, increased DMN intrusions during task performance, and diminished functional connectivity within the frontoparietal network (Lim and Dinges, 2010; Esposito et al., 2014). When the system is in such a suboptimal state, applying the same dual-site perturbation may not reinforce a task-positive configuration but instead push the system further from its optimal operating point, resulting in decreased performance. This is consistent with the broader principle that neuromodulation can have paradoxical or deleterious effects when the targeted circuit is not in a state that supports the intended modulation (Silvanto et al., 2018; Bradley et al., 2022).

The network sloppiness framework offers a complementary mechanistic interpretation. Chen et al. (2025) demonstrated that cognitive performance depends disproportionately on variations along stiff network dimensions, which represent the mechanistically influential axes of brain network activity. Within this framework, the DLPFC and mPFC are hypothesized to occupy dissociable positions along the stiff dimensions most relevant to WM control, although direct confirmation of this mapping would require concurrent neuroimaging during stimulation. Fatigue may reflect a shift of the brain state away from the stiff dimension that optimally supports WM, such that the system begins the task in a suboptimal region of network state space. Dual-site TMS, which perturbs activity along the DLPFC–mPFC dimension, would then nudge the system in opposite behavioral directions depending on whether it starts in a well-aligned (low-fatigue) or non-aligned (high-fatigue) configuration. Recent work on arousal-related cortical manifolds provides further context, showing that global brain dynamics fluctuate along low-dimensional manifolds linked to arousal and that these fluctuations shape cognitive readiness and responsiveness to external perturbation (Raut et al., 2025).

The WM load specificity of the observed effects is noteworthy and consistent with the idea that neuromodulation exerts its strongest leverage when the targeted neural networks are maximally recruited by the task demands (Silvanto et al., 2008; Luber and Lisanby, 2014). The 2-back condition places substantial demands on WM maintenance, updating, and inhibition, strongly engaging the DLPFC and requiring robust DMN suppression. Under these conditions, the dual-site perturbation has the greatest opportunity to influence the network balance that determines performance. In contrast, the 0-back condition imposes minimal WM load and may not sufficiently engage the DLPFC–mPFC circuit to be sensitive to the stimulation manipulation. Furthermore, the absence of reliable RT effects suggests that dual-site TMS selectively influenced the precision of WM representations rather than general response speed. Relatedly, the within-session stimulation order (iTBS-first vs. cTBS-first), which was counterbalanced between subjects, did not modulate the state-dependent pattern: a supplementary analysis replacing WM Load with TMS order in the three-way interaction confirmed that the bidirectional effect was qualitatively consistent regardless of whether excitatory or inhibitory stimulation was delivered first, consistent with the temporal overlap of iTBS and cTBS aftereffects during the task window.

Several limitations should be acknowledged. First, fatigue was assessed using a coarse binary self-report that does not capture the full complexity of underlying brain states. Although the Introduction outlines evidence that subjective fatigue ratings track meaningful variation in arousal and network dynamics, a continuous objective measure, such as pre-run pupillometry or frontal alpha asymmetry, would permit finer-grained modeling and could reveal whether the bidirectional pattern reflects a categorical state boundary or a graded modulation along a continuous arousal axis. Second, the absence of a pure sham condition (sham DLPFC + sham mPFC) means that we cannot determine whether the observed effects reflect absolute enhancement and impairment of WM accuracy or relative differences between two active protocols. Future studies should include a full sham condition to anchor stimulation effects to an unstimulated baseline. Third, no concurrent neuroimaging was collected, precluding direct assessment of how TMS altered DLPFC–mPFC connectivity or whether the stimulation shifted anticorrelation dynamics in the manner our mechanistic interpretation proposes. Combining dual-site TMS with concurrent fMRI or EEG in future studies would allow direct testing of these network-level mechanisms and help bridge the gap between behavioral outcomes and underlying neural changes. Finally, the sample size was modest, and all participants were healthy young adults. Generalizability to older adults, clinical populations with known prefrontal dysfunction, or alternative TMS parameters and target configurations remains to be established.

Despite these limitations, the findings carry important implications. Methodologically, they underscore the importance of accounting for momentary cognitive states in TMS studies. The state-dependent reversal observed here may help explain inconsistency in the WM–TMS literature, as studies not monitoring brain state fluctuations may average over opposing effects, yielding null results. Beyond momentary state, individual differences in psychological profiles and underlying cortical morphology, such as disorder-specific cortical thickness patterns (Yu et al., 2023) or distinct internalizing subtypes (Fan et al., 2023), may further shape susceptibility to neuromodulation and could inform the development of personalized stimulation strategies for clinical populations. Translationally, these findings support individualized, state-aware stimulation protocols in which real-time brain state assessments guide when and how to stimulate (Zrenner et al., 2026). Such closed-loop approaches could improve the reliability and efficacy of TMS-based cognitive interventions.

In conclusion, we demonstrate that adding inhibitory mPFC stimulation to excitatory DLPFC stimulation produces state-dependent changes in WM accuracy that are not observed with DLPFC stimulation alone, with the direction of modulation depending on the participant’s fatigue state. While the absence of a pure sham condition precludes definitive claims about absolute enhancement or impairment, the state-dependent dissociation observed here supports the broader principle that multi-site, network-sensitive stimulation approaches may capture dynamics that single-site protocols miss. These findings encourage future work incorporating full sham controls and objective state monitoring to further characterize the conditions under which dual-site prefrontal TMS can reliably modulate cognitive performance.

## Data Availability

The raw data supporting the conclusions of this article will be made available by the authors, without undue reservation.
